# Agreement and Repeatability of Three Fractional Exhaled Nitric Oxide Analyzers in Patients with Chronic Obstructive Pulmonary Disease: A Prospective Comparative Study

**DOI:** 10.3390/jcm15218744

**Published:** 2026-11-09

**Authors:** Claudio Candia, Silvestro Ennio D’Anna, Antonio Iovine, Carmen Lombardi, Claudia Merola, Mauro Maniscalco

**Affiliations:** 1 Istituti Clinici Scientifici Maugeri IRCCS, Pulmonary Rehabilitation Unit of Telese Terme Institute, 82037 Telese Terme, Italy; claudio.candia@icsmaugeri.it (C.C.); silvestro.danna@icsmaugeri.it (S.E.D.); antonio.iovine@icsmaugeri.it (A.I.); carmen.lombardi@icsmaugeri.it (C.L.); claudia.merola@icsmaugeri.it (C.M.); 2 Department of Advanced Biomedical Sciences, University of Naples “Federico II”, 80126 Naples, Italy

**Keywords:** fractional exhaled nitric oxide, FeNO, chronic obstructive pulmonary disease, COPD, reproducibility, biomarker

## Abstract

**Background and aim:** Fractional exhaled nitric oxide (FeNO) is a non-invasive biomarker of airway inflammation that may provide complementary information on inflammatory phenotypes in chronic obstructive pulmonary disease (COPD). The increasing availability of portable FeNO analyzers requires assessment of their reproducibility and agreement before measurements obtained with different devices can be considered interchangeable. In this study, we aimed to compare the performance of three FeNO analyzers in patients with COPD. **Methods:** In this prospective study, consecutive patients with COPD underwent FeNO assessment using three analyzers: Medisoft Hyp’Air FeNO, Sunvou CA2122, and Sunvou TM2120. Two consecutive valid measurements were obtained with each device. Both within-device repeatability and between-device agreement were evaluated, while the number of measurement attempts recorded for each analyzer was investigated as an exploratory analysis of feasibility. **Results:** In total, 56 participants (69.6% male), with a mean age of 70.8 ± 4.3 years and a mean FEV1 of 42.3 ± 13.1% predicted, were enrolled. All analyzers showed excellent within-device repeatability, with ICCs of 0.976 for Hyp’Air, 0.967 for CA2122, and 0.992 for TM2120. Between-device absolute agreement was highest for Hyp’Air versus TM2120 (ICC 0.989), followed by CA2122 versus TM2120 (ICC 0.943) and Hyp’Air versus CA2122 (ICC 0.931). The median number of attempts was 4.5 [3.8–5.0] with Hyp’Air, 3.0 [2.0–3.0] with CA2122, and 2.0 [2.0–3.0] with TM2120 (overall *p* < 0.001). **Conclusions:** All three FeNO analyzers demonstrated excellent within-device repeatability in patients with COPD. TM2120 showed closer agreement with Hyp’Air than CA2122, although the limited representation of elevated FeNO values prevents firm conclusions regarding inter-device agreement at higher concentrations.

## 1. Introduction

Fractional exhaled nitric oxide (FeNO) is a non-invasive biomarker reflecting nitric oxide production within the respiratory tract [1]. Its measurement is rapid, repeatable, and does not require biological sampling, making it particularly suitable for repeated assessment in both clinical practice and respiratory research [2]. Standardized recommendations for FeNO measurement and interpretation have facilitated its incorporation into the assessment of inflammatory airway diseases [3].

The clinical role of FeNO is most established in asthma, where increased levels are associated with type 2 and eosinophilic airway inflammation and may contribute to disease phenotyping, prediction of corticosteroid responsiveness, and treatment monitoring [4]. Nevertheless, eosinophilic airway inflammation is not restricted to asthma. COPD is increasingly recognized as a heterogeneous disorder comprising multiple inflammatory phenotypes, and a subgroup of patients may exhibit eosinophilic or type 2 inflammatory features [5]. In these patients, FeNO has been investigated as a potential non-invasive biomarker of airway eosinophilia and corticosteroid responsiveness [6,7]. Although its role in COPD is less established than in asthma, previous evidence suggests that FeNO may have potential value for identifying selected inflammatory phenotypes and monitoring treatment response [8]. In particular, corticosteroid administration may result in greater FeNO reductions in COPD patients with higher baseline FeNO concentrations, supporting the biological relevance of the marker in at least a subset of patients [9]. At the same time, interpretation of FeNO in COPD remains challenging because measurements may be influenced by smoking exposure, inhaled corticosteroid treatment, airway obstruction, exacerbation status, and other biological sources of variability [8].

An essential prerequisite for any biomarker intended for longitudinal clinical use is adequate analytical reproducibility. Previous studies have demonstrated that FeNO can be measured reproducibly in patients with COPD, both over short intervals and using portable electrochemical analyzers [10,11]. However, the reproducibility of a single instrument does not necessarily imply interchangeability between different analyzers. In fact, previous comparative studies demonstrated that FeNO measurements obtained using different commercially available analyzers cannot invariably be considered interchangeable [12,13].

In particular, Molino et al. directly compared three FeNO analyzers in healthy subjects and patients with asthma or COPD and reported good overall relationships between instruments but also device-dependent differences in absolute measurements [14]. These observations emphasize that assessment of new devices should include not only correlation but also repeatability and absolute agreement analyses, such as intraclass correlation coefficients and Bland–Altman methods.

More recently, the potential clinical relevance of FeNO in COPD has been further highlighted by the introduction of biological therapies targeting type 2 inflammation. Post-hoc analyses of randomized trials of dupilumab have suggested that higher baseline FeNO concentrations may identify patients deriving greater treatment benefit, while FeNO reductions during treatment reflect modulation of type 2 inflammatory activity [15]. In addition, a recent prospective multicenter study by Freund et al. [16] reported a significant reduction in FeNO following dupilumab initiation and an association between higher baseline FeNO levels and improvements in oscillometry-defined small airway dysfunction. Although these findings require further validation, they highlight the growing interest in FeNO as a complementary biomarker for treatment stratification and longitudinal assessment in selected patients with COPD.

As the potential clinical applications of FeNO in COPD expand, ensuring the reliability and comparability of measurements obtained with different analyzers becomes increasingly relevant. While the analytical characteristics of FeNO analyzers are primarily determined by device-specific technology and measurement protocols, the interpretation of inter-device agreement also depends on the range of values investigated [13,17]. In COPD, smoking exposure and inhaled corticosteroid treatment may influence FeNO concentrations [18], making it relevant to establish agreement across the measurement range encountered in this population. Furthermore, the evaluation of measurement feasibility may provide complementary information on the practical use of these devices in routine respiratory care. Therefore, the present study aimed to assess the repeatability, inter-device agreement, and technical feasibility of three FeNO analyzers in a clinical cohort of patients with COPD.

## 2. Patients and Methods

### 2.1. Study Design and Population

This was a prospective, single-center, cross-sectional method-comparison study with a repeated-measures design, conducted at the Pulmonary Rehabilitation Unit of Istituti Clinici Scientifici Maugeri IRCCS (Telese Terme, Italy) between July 2025 and July 2026.

Consecutive patients with a confirmed diagnosis of COPD were considered eligible for inclusion. COPD was diagnosed according to established clinical and spirometric criteria [19]. Exclusions were applied to individuals with a recent (within the previous 30 days) exacerbation of COPD; individuals with a concomitant diagnosis of asthma or allergic rhinitis; and individuals with a concomitant or recent (within the previous 30 days) viral infection.

The study was conducted in accordance with the Declaration of Helsinki and reported according to the Strengthening the Reporting of Observational Studies in Epidemiology (STROBE) guidelines [20]. The study protocol was reviewed and approved by the Comitato Etico Territoriale Campania 1 (approval no. 3/24 Maugeri del Registro). Written informed consent was obtained from all participants.

### 2.2. Study Endpoints

The primary endpoint of the study was to assess within-device repeatability and between-device absolute agreement of FeNO measurements obtained with the three analyzers. Within-device repeatability was evaluated by comparing two consecutive valid measurements performed with each device, whereas between-device agreement was primarily assessed by comparing CA2122 and TM2120 with Hyp’Air. The latter was selected as the reference comparator based on its previous use and characterization in published method-comparison studies involving patients with chronic respiratory diseases, including COPD [14]. This approach allowed the agreement of the two Sunvou analyzers to be evaluated against a previously investigated FeNO measurement system.

Secondary analyses included the agreement between the two portable analyzers, CA2122 and TM2120, and the assessment of systematic and proportional inter-device bias across the observed FeNO range using Bland–Altman analysis.

Exploratory endpoints included the evaluation of agreement between Hyp’Air and CA2122 after restricting the analysis to lower FeNO ranges in order to investigate the influence of higher FeNO concentrations on proportional disagreement. In addition, the number of attempts required to obtain valid FeNO measurements with each analyzer was assessed as an exploratory indicator of technical feasibility.

### 2.3. Fractional Exhaled Nitric Oxide Measurement

FeNO was measured using three commercially available analyzers: Medisoft Hyp’Air FeNO (Medisoft, Sorinnes, Belgium), Sunvou CA2122 (Sunvou Medical Electronics, Wuxi, China), and Sunvou TM2120 (Sunvou Medical Electronics, Wuxi, China). The main technical and operational characteristics of the FeNO analyzers are summarized in Supplemental Table S1.

Measurements were performed with participants seated and according to standardized recommendations for FeNO assessment [3]. Patients were instructed to refrain from eating and smoking for at least 2 h before FeNO assessment and to avoid physical exercise and bronchodilator use for 12 h prior to the procedure. Briefly, participants performed a controlled expiration at a target flow rate of 50 mL/s with the required exhalation duration determined by the manufacturer-specific measurement protocol. Two valid FeNO measurements were obtained with each analyzer, and the mean of the two measurements was used for between-device comparisons. All analyzers underwent calibration and quality control procedures in accordance with the respective manufacturers’ recommendations, including verification using certified nitric oxide calibration gas. The measurement order was determined for each participant using a randomly generated numerical sequence. An operational interval of approximately 10 min was allowed between measurements performed with different analyzers. All measurements were conducted by the same trained operator (C.C.) according to the standardized study protocol. For each analyzer, the total number of attempts required to obtain two valid FeNO measurements was recorded. An attempt was defined as each expiratory maneuver initiated with a device, regardless of whether it yielded a valid FeNO measurement. Successful attempts were defined as those resulting in a valid FeNO measurement, indicated by the display of a FeNO value for the portable analyzers (CA2122 and TM2120) and by a FeNO value associated with an instrument-reported confidence level ≥ 95% for Hyp’Air. Attempts were classified as unsuccessful when the expiratory maneuver failed to meet the device-specific acceptance criteria, including unstable expiratory flow, insufficient exhalation duration, or an unacceptable expiratory flow curve. However, the specific reasons for unsuccessful attempts were not systematically recorded according to device and therefore could not be quantitatively compared among analyzers.

### 2.4. Statistical Analysis

Continuous variables were assessed for distributional characteristics and are reported as mean ± standard deviation (SD) or median and interquartile range [IQR], as appropriate. Categorical variables are presented as absolute numbers and percentages.

No formal a priori sample size calculation was performed, as reliable preliminary estimates of the variability of paired differences between the investigated analyzers in patients with COPD were unavailable. Accordingly, no retrospective power calculation was used to infer adequacy of the sample size; instead, the precision achieved with the available sample was quantified through 95% confidence intervals around each Bland–Altman limit of agreement. Therefore, consecutive eligible patients were enrolled during the study period, and the precision of the agreement estimates was evaluated by calculating 95% confidence intervals around the Bland–Altman limits of agreement.

Within-device repeatability was evaluated by comparing the two consecutive valid FeNO measurements obtained with each analyzer. Repeatability was quantified using a two-way random-effects, absolute-agreement, single-measure intraclass correlation coefficient [ICC(A,1), equivalent to ICC(2,1)]. ICC values were interpreted according to conventional thresholds, with values < 0.50 indicating poor reliability, 0.50–0.75 moderate reliability, 0.75–0.90 good reliability, and >0.90 excellent reliability.

For inter-device analyses, the mean of the two valid measurements obtained with each analyzer was calculated for each participant. Hyp’Air was considered the reference comparator, and the primary agreement analyses therefore included Hyp’Air versus CA2122 and Hyp’Air versus TM2120. Agreement between CA2122 and TM2120 was additionally evaluated as a secondary analysis. Inter-device absolute agreement was quantified using ICC(A,1), with 95% confidence intervals estimated by bootstrap resampling.

Agreement was further assessed using the Bland–Altman method. For each device pair, the mean paired difference was calculated as an estimate of systematic bias, together with the corresponding 95% limits of agreement (LoAs), defined as the mean difference ±1.96 standard deviations of the paired differences. Approximate 95% confidence intervals for each LoA were calculated using the standard error of the agreement limits and the t distribution with n − 1 degrees of freedom, according to the method described by Bland and Altman [21]. Potential proportional bias was investigated by linear regression of the inter-device differences against the corresponding mean FeNO values. A statistically significant regression slope was interpreted as evidence that the magnitude of disagreement varied across the measurement range.

Because visual inspection of the Bland–Altman plots suggested greater dispersion at higher FeNO concentrations, post-hoc exploratory sensitivity analyses were performed for the Hyp’Air versus CA2122 comparison. The thresholds of ≤40 ppb and ≤30 ppb were selected descriptively based on the observed distribution rather than as prespecified clinical cut-offs: only one participant had Hyp’Air FeNO > 40 ppb, and six had values > 30 ppb. These analyses were intended to assess the influence of sparsely represented higher FeNO observations on the estimated proportional-bias slope and limits of agreement.

As an exploratory measure of technical feasibility, the number of attempts required to obtain valid FeNO measurements with each analyzer was recorded and compared across devices. Because these data were paired and not normally distributed, differences among the three analyzers were assessed using the Friedman test. When the overall test was statistically significant, post hoc pairwise comparisons were performed using Wilcoxon signed-rank tests. To account for multiple pairwise comparisons, adjusted *p* values were calculated using the Bonferroni method. The number of attempts is reported as median [interquartile range]. All tests were two-sided, and a *p* value < 0.05 was considered statistically significant. Statistical analyses were performed using RStudio version 2023.9.1.494 (Posit team (2023); RStudio: Integrated Development Environment for R, Posit Software, PBC, Boston, MA, USA. URL: http://www.posit.co/).

### 2.5. Use of Artificial Intelligence Tools

During the preparation of this manuscript, the authors used artificial intelligence tools solely for English language polishing and stylistic refinement. These tools were not used for data analysis, interpretation of results, generation of scientific content, or formulation of conclusions. All scientific content was critically reviewed and approved by the authors, who take full responsibility for the final manuscript.

## 3. Results

### 3.1. Study Population

A total of 72 individuals were screened for inclusion. Of them, 5 presented with a history of asthma, 7 presented with concomitant allergic rhinitis, and 4 refused participation in the protocol. Therefore, a total of 56 patients with COPD were included in the final analysis. The mean age was 70.8 ± 4.3 years; 39 participants (69.6%) were male, while the mean FEV_1_ was 42.3 ± 13.1% predicted, indicating a population characterized by clinically relevant airflow obstruction (Table 1).

### 3.2. Within-Device Repeatability

All three FeNO analyzers showed an excellent agreement between consecutive measurements (Table 2). In detail, for Hyp’Air, the ICC for repeated measurements was 0.976 (bootstrap 95% CI 0.950–0.985). The corresponding ICC was 0.967 (95% CI 0.927–0.982) for CA2122 and 0.992 (95% CI 0.979–0.996) for TM2120. The mean difference between the first and second measurements was −0.89 ppb for Hyp’Air, −0.86 ppb for CA2122, and −0.23 ppb for TM2120. The corresponding Bland–Altman limits of agreement were −4.88 to 3.09 ppb, −4.89 to 3.18 ppb, and −2.63 to 2.16 ppb, respectively. Descriptively, TM2120 showed the narrowest within-device limits of agreement between consecutive measurements.

### 3.3. Between-Device Agreement of FeNO Measurements

**Figure 1:**
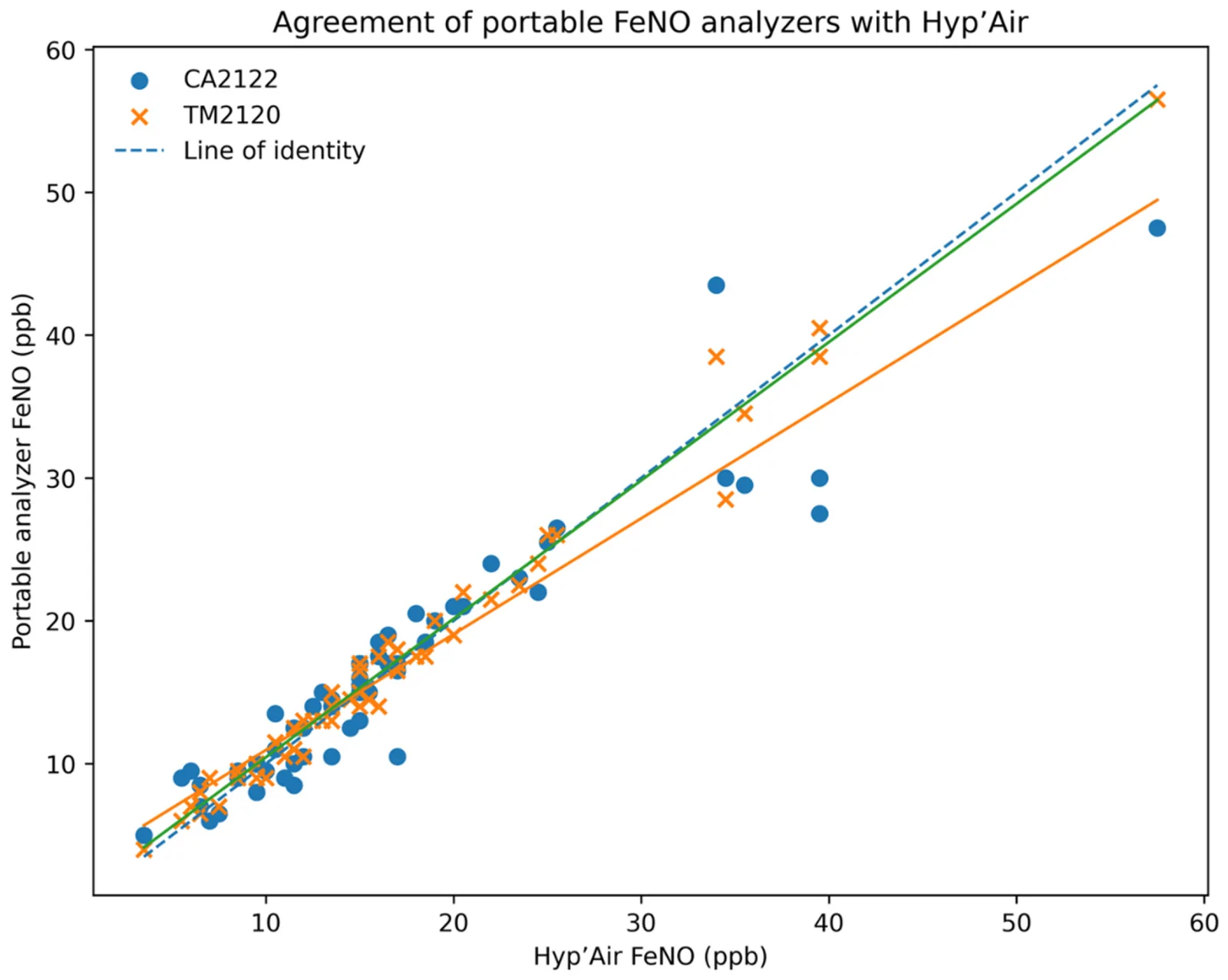
Agreement of portable FeNO analyzers with Hyp’Air. Scatter plot showing mean FeNO values obtained with CA2122 and TM2120 plotted against the corresponding mean Hyp’Air measurements. Each point represents one participant. The dashed line represents the line of identity (y = x), while fitted regression lines illustrate the relationship between Hyp’Air and each portable analyzer. TM2120 measurements closely followed the identity line across the observed FeNO range, whereas greater dispersion was observed for CA2122 at higher FeNO concentrations. FeNO, fractional exhaled nitric oxide. Coloured lines represent fitted regression lines.

**Figure 2:**
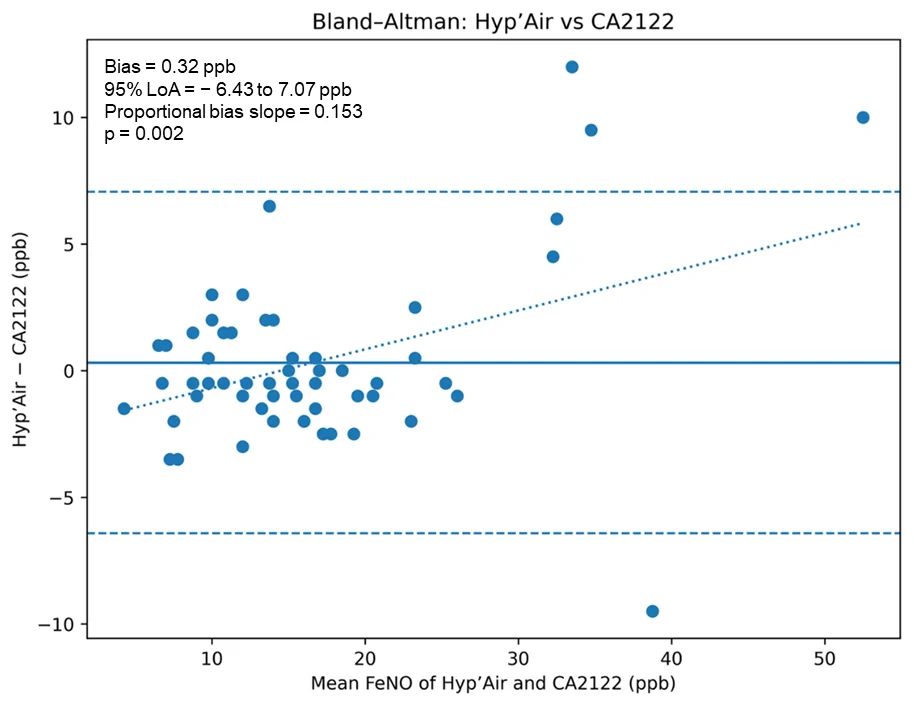
Agreement between Hyp’Air and CA2122. The mean bias was +0.32 ppb, with 95% limits of agreement from −6.43 to 7.07 ppb. Linear regression of the paired differences against the mean FeNO concentration demonstrated significant proportional bias (slope 0.153, *p* = 0.002), with increasing disagreement at higher FeNO values. The solid horizontal line indicates the mean bias, dashed horizontal lines indicate the 95% limits of agreement, and the dotted line represents the fitted regression of inter-device differences on mean FeNO values. FeNO, fractional exhaled nitric oxide; ppb, parts per billion.

**Figure 3:**
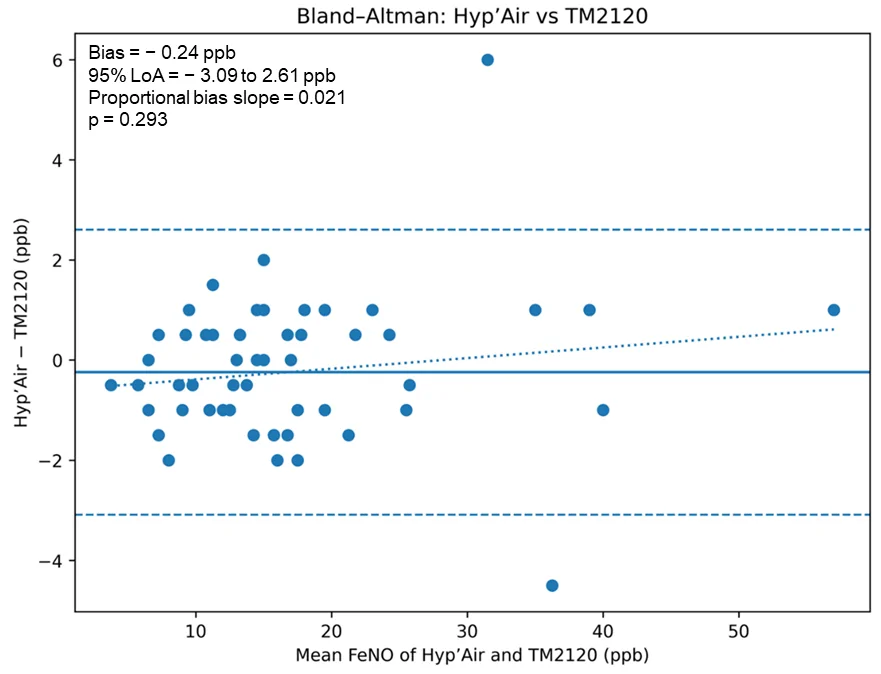
Agreement between Hyp’Air and TM2120. The mean bias was −0.24 ppb, with 95% limits of agreement from −3.09 to 2.61 ppb. No significant proportional bias was observed (slope 0.021, *p* = 0.293). The solid horizontal line indicates the mean bias, dashed horizontal lines indicate the 95% limits of agreement, and the dotted line represents the fitted regression of inter-device differences on mean FeNO values. FeNO, fractional exhaled nitric oxide; ppb, parts per billion.

Between-device agreement was high for all comparisons but differed quantitatively between analyzer pairs, as observed in Figure 1. The strongest agreement was observed between Hyp’Air and TM2120, with an ICC of 0.989 (95% CI 0.974–0.995) (Table 3). No significant proportional bias was observed for the Hyp’Air–TM2120 comparison (*p* = 0.293). Agreement between Hyp’Air and CA2122 remained high (Figure 2), although lower than for Hyp’Air versus TM2120 (Figure 3). The mean difference was 0.32 ppb, with wider limits of agreement ranging from −6.43 to 7.07 ppb. Despite the small overall mean bias, regression analysis demonstrated a significant relationship between the magnitude of the difference and the mean FeNO concentration (*p* = 0.002), suggesting proportional bias. Similarly, CA2122 and TM2120 showed high overall agreement (ICC 0.943, 95% CI 0.894–0.969), as shown in Supplemental Figure S1. The mean difference was −0.56 ppb, with limits of agreement from −6.59 to 5.46 ppb. Evidence of proportional bias was again observed (*p* = 0.003).

### 3.4. Exploratory Analysis of Agreement at Higher FeNO Concentrations

Inspection of the Bland–Altman plot suggested that the proportional disagreement observed between Hyp’air and CA2122 was concentrated primarily at higher FeNO concentrations. Only 6 of the 56 participants (10.7%) had Hyp’air FeNO values > 30 ppb, while only one participant had a value > 40 ppb. After exclusion of the single participant with Hyp’Air FeNO > 40 ppb, the proportional-bias slope decreased from 0.153 to 0.106 (*p* = 0.060). When the analysis was restricted to participants with Hyp’Air FeNO ≤ 30 ppb (n = 50), the estimated slope was −0.023 (*p* = 0.666), while the 95% limits of agreement narrowed from −6.43 to 7.07 ppb in the overall cohort to −3.97 to 3.39 ppb (Supplemental Table S2). Given the small number of observations in the upper FeNO range represented in this cohort, these findings should be considered descriptive and exploratory rather than evidence of a definitive concentration-dependent device effect.

### 3.5. Number of Measurement Attempts

**Figure 4:**
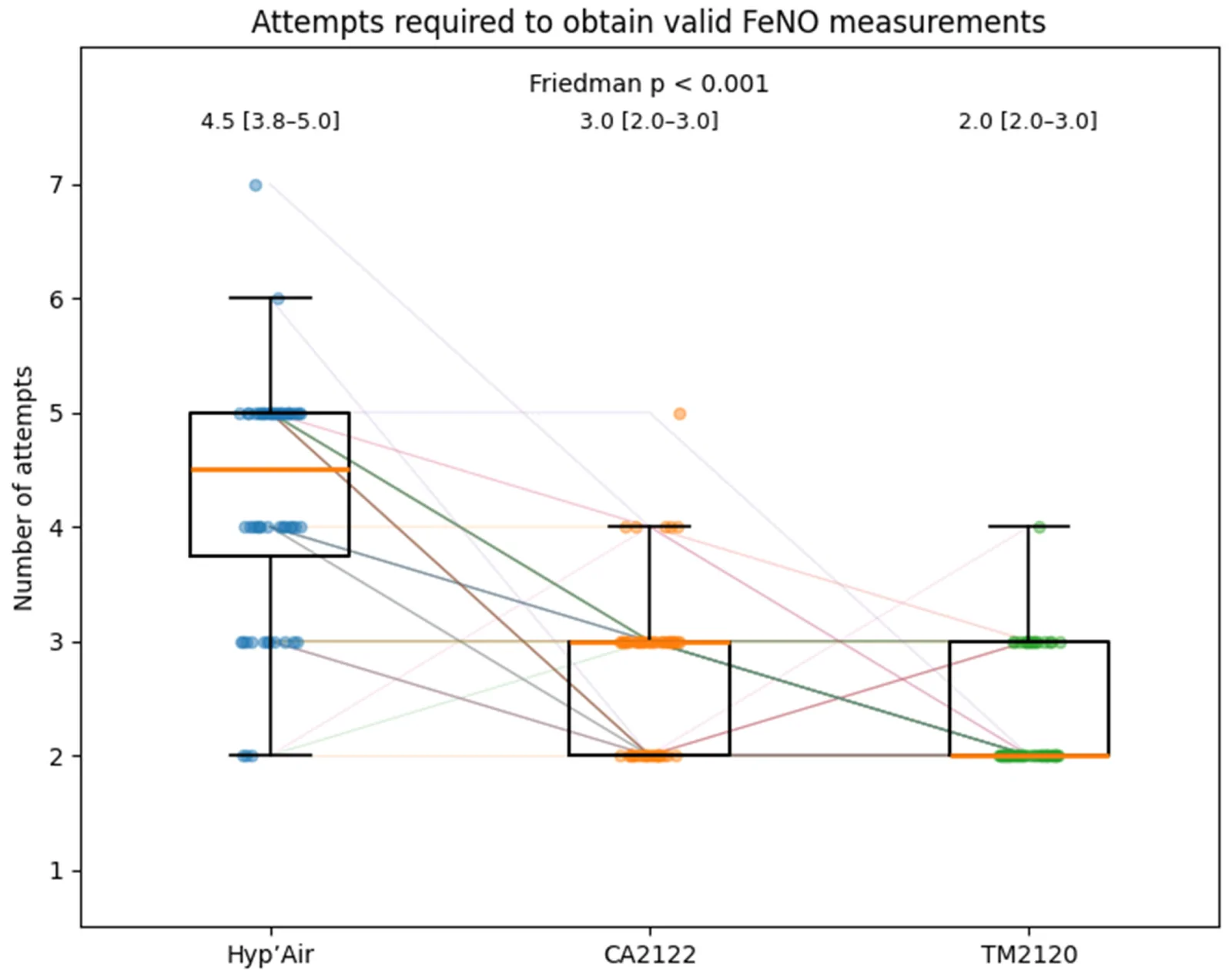
Number of attempts required to obtain valid FeNO measurements with each analyzer. The median number of attempts was 4.5 [3.8–5.0] for Hyp’Air, 3.0 [2.0–3.0] for CA2122, and 2.0 [2.0–3.0] for TM2120. Individual paired observations are shown, with lines connecting measurements obtained in the same participant; boxplots represent the median and interquartile range. The overall difference among analyzers was significant (Friedman *p* < 0.001). FeNO, fractional exhaled nitric oxide.

The number of attempts required to obtain valid FeNO measurements differed significantly among the three analyzers (Friedman *p* < 0.001), as shown in Figure 4. The median number of attempts was 4.5 [3.8–5.0] with Hyp’Air, 3.0 [2.0–3.0] with CA2122, and 2.0 [2.0–3.0] with TM2120. Post hoc pairwise comparisons showed that both portable analyzers required significantly fewer attempts than Hyp’Air. In particular, the number of attempts was lower with CA2122 compared with Hyp’Air (*p* < 0.001) and with TM2120 compared with Hyp’Air (*p* < 0.001). Moreover, TM2120 required significantly fewer attempts than CA2122 (*p* = 0.004).

## 4. Discussion

Our study showed that all three evaluated FeNO analyzers presented with excellent within-device repeatability in patients with COPD, with intraclass correlation coefficients exceeding 0.96. Although overall between-device agreement was high, relevant differences emerged when absolute agreement was examined. Hyp’Air and TM2120 showed the closest agreement, with minimal systematic bias and relatively narrow Bland–Altman limits of agreement (−3.09 to +2.61 ppb). Conversely, comparisons involving CA2122 showed wider limits of agreement and evidence of proportional bias. These findings are consistent with previous studies showing that high correlations between FeNO analyzers do not necessarily imply their clinical interchangeability [12,13,14].

From a practical perspective, all three devices provided highly repeatable FeNO measurements under standardized conditions, supporting their potential use in clinical respiratory settings. Nevertheless, the observed differences in inter-device agreement should be considered when interpreting measurements obtained with different instruments. In particular, differences of several ppb could be relevant when assessing modest longitudinal changes or measurements approaching clinically meaningful thresholds. Although no universally accepted maximum clinically allowable difference between FeNO analyzers has been established, our findings suggest that serial measurements should preferably be performed using the same device, especially when FeNO is used to monitor changes over time. The closer agreement observed between Hyp’Air and TM2120 is reassuring within the measurement range represented in our cohort but does not establish unrestricted interchangeability between these analyzers.

The clinical relevance of reliable FeNO assessment in COPD is becoming increasingly apparent with the introduction of biological therapies targeting type 2 inflammation. In particular, recent evidence suggests that FeNO may provide complementary information regarding response to dupilumab. Freund et al. [16], in a prospective multicenter study, reported an association between higher baseline FeNO concentrations and improvements in oscillometry-defined small airway dysfunction following dupilumab initiation, together with reductions in FeNO values during treatment. These observations highlight the potential value of FeNO for identifying and longitudinally monitoring selected patients with COPD receiving biological therapies. In this context, consistency of measurement becomes particularly important, since device-related variability could complicate the interpretation of treatment-associated changes.

Beyond analytical agreement, practical differences between devices may also influence their use in routine care. Both portable devices were associated with a lower number of recorded attempts than Hyp’Air. This difference was most pronounced with TM2120, for which the median number of attempts was two. The ability to obtain reliable FeNO measurements with fewer maneuvers could be advantageous in elderly individuals and in patients with severe airflow obstruction, respiratory muscle weakness, or reduced exercise tolerance. It could also improve workflow in outpatient respiratory clinics and pulmonary rehabilitation settings. However, the present study was not designed as a formal usability assessment, and the observed differences cannot be attributed solely to greater ease of use. In particular, device-specific measurement protocols and acceptance criteria differed across analyzers and may have contributed to the number of attempts required to obtain two valid measurements. Dedicated studies incorporating measurement duration, failure mechanisms, operator and patient experience, and validated usability outcomes are needed to formally compare device usability.

Finally, the characteristics of the present cohort should also be considered when interpreting these findings. Most participants had moderate-to-very-severe airflow obstruction, 92.9% were receiving inhaled corticosteroids, and 55.4% were active smokers. Both smoking and corticosteroid exposure may influence FeNO levels [1] and may have contributed to the predominance of low-to-intermediate FeNO values observed in this study. More broadly, COPD is characterized by substantial heterogeneity in inflammatory traits, respiratory mechanics, gas exchange, and ventilatory control [22]. Accordingly, the present findings may be most applicable to clinical COPD populations with characteristics similar to those of the present cohort and should not necessarily be extrapolated to the full spectrum of COPD encountered in routine practice.

Importantly, our findings concern measurement performance rather than the clinical utility of FeNO-guided management in COPD, as our study does not establish whether FeNO-based therapeutic decisions improve clinical outcomes, which remains a separate question requiring prospective outcome-oriented studies.

The present study has several strengths. First, measurements were obtained in a well-characterized clinical COPD cohort with a broad degree of airflow obstruction. Second, two consecutive measurements were available for each analyzer, allowing separate assessment of within-device repeatability and between-device agreement. Third, agreement was evaluated using complementary statistical approaches rather than relying exclusively on correlation coefficients. Finally, the recording of the number of measurement attempts provides preliminary information regarding practical feasibility.

Several limitations should also be acknowledged. First, this was a single-center study involving a relatively limited sample size. Second, most participants had FeNO concentrations in the low-to-intermediate range, and only a small proportion had elevated FeNO values. Therefore, the agreement estimates are most robust within the measurement range predominantly represented in this cohort, whereas findings concerning higher FeNO concentrations should be considered exploratory and require confirmation in populations including a larger number of patients with elevated FeNO values. This consideration may be particularly relevant to the proportional bias observed for CA2122. Third, the number of attempts was an exploratory measure and cannot substitute for a validated evaluation of device usability. Moreover, although the overall number of attempts was prospectively recorded, the specific reasons for unsuccessful maneuvers were not systematically categorized according to device. In addition, the relatively narrow age distribution of our cohort limits the generalizability of our findings to younger patients or populations with a broader age range, although no age-related differences in device performance can be inferred from the present study. Finally, our findings should not be extrapolated to other clinical populations or to measurements obtained under different technical conditions without external validation.

## 5. Conclusions

The three FeNO analyzers demonstrated excellent within-device repeatability in this clinical COPD cohort, supporting their use for FeNO assessment under standardized conditions. TM2120 showed particularly close agreement with Hyp’Air within the FeNO range represented in the study, with minimal systematic bias and relatively narrow limits of agreement. Comparisons involving CA2122 showed wider limits of agreement and greater dispersion in the upper FeNO range represented in this cohort; however, the limited number of participants with elevated FeNO makes this observation exploratory. The portable analyzers required fewer recorded attempts to obtain valid measurements, although differences in device-specific technical protocols and acceptance criteria preclude interpreting this finding as evidence of superior usability. In daily clinical practice, all three devices may provide repeatable measurements, but longitudinal FeNO monitoring should preferably be performed using the same analyzer to minimize instrument-related variability. These considerations may become particularly relevant as FeNO assessment gains importance in selected patients with COPD receiving biological therapies. Further studies involving more heterogeneous populations and higher FeNO concentrations are warranted before broader conclusions regarding device interchangeability can be drawn.

## Figures and Tables

**Table 1:** Clinical characteristics of the study population. Data are presented as mean ± SD, median [IQR], or n (%) as appropriate.

Variable	Overall Population (n = 56)
Age, years	70.8 ± 4.3
Males, n (%)	39 (69.6)
Body Mass Index, kg/m^2^	24.8 ± 3.7
Active smokers, n (%)	31 (55.4)
Smoking exposure, pack/years	29.2 ± 9.8
ICS users, n (%)	52 (92.9)
Exacerbation rate, n/year	2.0 [1.0–2.0]
Blood eosinophils, cells/mm^3^	150.0 [75.0–230.0]
GOLD spirometric grade II, n (%)	14 (25.0)
GOLD spirometric grade III, n (%)	31 (55.4)
GOLD spirometric grade IV, n (%)	11 (19.6)
FEV_1_, % predicted	42.3 ± 13.1
FEV1/FVC ratio	0.49 ± 0.09
Hyp’Air FeNO, ppb	15.0 [10.9–18.6]
CA2122 FeNO, ppb	15.0 [10.0–20.1]
TM2120 FeNO, ppb	14.8 [10.9–18.6]

**Table 2:** Within-device repeatability of consecutive FeNO measurements obtained with each analyzer.

Analyzer	ICC(A,1) (95% CI)	Mean Difference, ppb	Lower LoA (95% CI)	Upper LoA (95% CI)
Hyp’Air	0.976 (0.950–0.985)	−0.89	−4.88 (−5.81, −3.94)	+3.09 (+2.16, +4.03)
CA2122	0.967 (0.927–0.982)	−0.86	−4.89 (−5.84, −3.94)	+3.18 (+2.23, +4.12)
TM2120	0.992 (0.979–0.996)	−0.23	−2.63 (−3.19, −2.06)	+2.16 (+1.60, +2.72)

ICC, intraclass correlation coefficient; CI, confidence interval; LoA, limit of agreement; FeNO, fractional exhaled nitric oxide.

**Table 3:** Agreement between devices.

Comparison	ICC(A,1) (95% CI)	Mean Bias, ppb	Lower LoA (95% CI), ppb	Upper LoA (95% CI), ppb	Proportional-Bias Slope	*p* Value
**Hyp’Air vs. CA2122**	0.931 (0.892–0.955)	0.32	−6.43 (−8.01 to −4.84)	7.07 (5.48 to 8.65)	0.153	**0.002**
**Hyp’Air vs. TM2120**	0.989 (0.974–0.995)	−0.24	−3.09 (−3.76 to −2.42)	2.61 (1.94 to 3.27)	0.021	0.293
**CA2122 vs. TM2120**	0.943 (0.894–0.969)	−0.56	−6.59 (−8.01 to −5.17)	5.46 (4.05 to 6.88)	−0.131	**0.003**

ICC, intraclass correlation coefficient; CI, confidence interval; LoA, limit of agreement; ppb, parts per billion.

## Data Availability

The data supporting this study’s findings are available from the corresponding author upon reasonable request.

## Supplementary Materials

The following supporting information can be downloaded at: https://www.mdpi.com/article/10.3390/jcm15218744/s1; Table S1: Comparison of the main technical features of the FeNO analyzers; Table S2: Exploratory sensitivity analysis of agreement between Hyp’Air and CA2122 after progressively restricting the upper FeNO range; Figure S1: Agreement between Sunvou CA2122 and TM2120.
