# Supplementary material for: Agreement and Repeatability of Three Fractional Exhaled Nitric Oxide Analyzers in Patients with Chronic Obstructive Pulmonary Disease: A Prospective Comparative Study
Source: J Clin Med. 2026 Nov 9;15(21):8744. doi: 10.3390/jcm15218744 (PMC13764312; doi:10.3390/jcm15218744)
Supplement: Supplementary file 1 [file jcm-15-08744-s001.zip › jcm-4610024-supplementary.pdf]

Supplemental Material

Agreement and Repeatability of Three Fractional Exhaled Nitric Oxide Analyzers in Patients with Chronic Obstructive Pulmonary Disease: A Prospective Comparative Study

Claudio Candia[1], Silvestro Ennio D'Anna[1], Antonio Iovine[1], Carmen Lombardi[1], Claudia Merola[1]

Mauro Maniscalco[1,2]

[1]Istituti Clinici Scientifici Maugeri IRCCS, Pulmonary Rehabilitation Unit of Telese Terme Institute, 82037 Telese Terme, Italy

[2]Department of Advanced Biomedical Sciences, University of Naples "Federico II", 80126 Naples, Italy

Supplemental Table S1 – Comparison of the main technical features of the FeNO analyzers.

Characteristic	Medisoft Hyp'Air FeNO	Sunvou CA2122	Sunvou TM2120
Sampling method	Controlled single-breath maneuver with pseudo-online sampling and internal sample collection	Online, offline and tidal-breathing sampling options	Online single-breath sampling
Expiratory flow for FeNO	50 mL/s	50 mL/s	50 mL/s
Required exhalation duration for FeNO	10 s	≥6 s in adults	10 s
NO sensor technology	Electrochemical NO sensor	Nano Coulomb® electrochemical NO sensor	Nano Coulomb® electrochemical NO sensor
Maneuver acceptance criteria	Software-guided maneuver with real-time expiratory flow and mouth-pressure monitoring and quality-control indicators	Device-guided expiratory maneuver with controlled flow and sufficient exhalation duration; valid FeNO value displayed following analysis	Continuous expiration without air leakage, maintenance of the visual flow indicator within the target zone and successful completion of breath sampling
Interval between repeated measurements	Not specified in the manufacturer documentation consulted	Not specified in the manufacturer documentation consulted	Not specified in the manufacturer documentation consulted
Calibration/QC procedures	Manufacturer-recommended NO sensor calibration every six months; software-based quality control	Self-calibration, gas calibration and device/user quality-control procedures	Self-calibration, gas calibration and automatic QC upon measurement initiation

Supplemental Table S2 - Exploratory sensitivity analysis of agreement between Hyp'Air and CA2122 after progressively restricting the upper FeNO range.

Analysis	n	ICC(A,1) (95% CI)	Mean bias, ppb	95% limits of agreement, ppb	Proportional-bias slope	p value
Overall cohort	56	0.931 (0.892–0.955)	0.32	−6.43 to 7.07	0.153	0.002
Hyp'Air FeNO ≤40 ppb	55	0.919 (0.883–0.949)	0.15	−6.15 to 6.44	0.106	0.060
Hyp'Air FeNO ≤30 ppb	50	0.937 (0.884–0.965)	−0.29	−3.97 to 3.39	−0.023	0.666

Supplemental Figure S1 – Agreement between Sunvou CA2122 and TM2120.

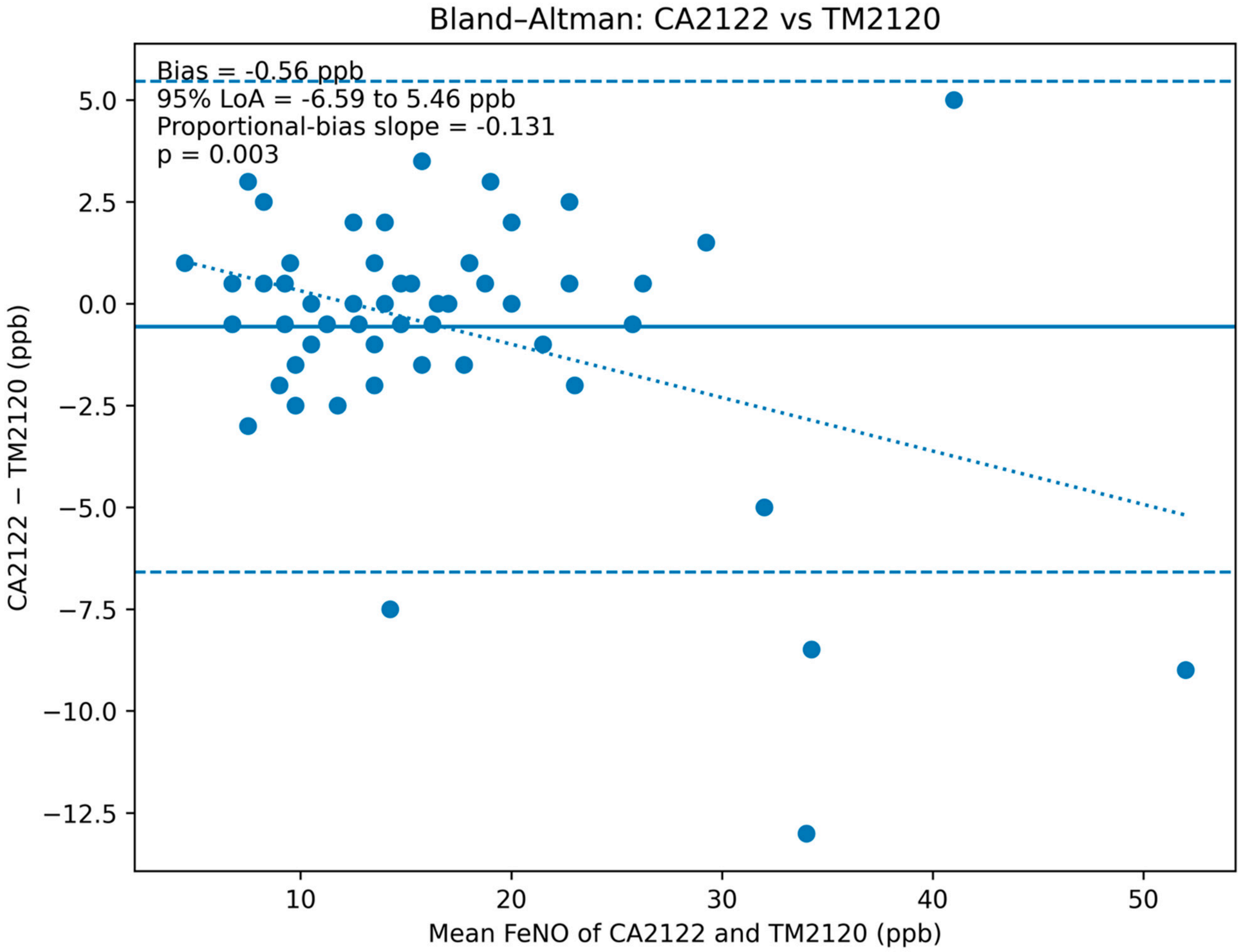

Bland–Altman: CA2122 vs TM2120
Bias = -0.56 ppb
95% LoA = -6.59 to 5.46 ppb
Proportional-bias slope = -0.131
p = 0.003
CA2122 − TM2120 (ppb)
Mean FeNO of CA2122 and TM2120 (ppb)
5.0
2.5
0.0
−2.5
−5.0
−7.5
−10.0
−12.5
10
20
30
40
50
